# Editorial: Nutritional requirements in production animals

**DOI:** 10.3389/fvets.2023.1138636

**Published:** 2023-02-07

**Authors:** Polyana Pizzi Rotta, Marcos Inácio Marcondes, Terry Engle

**Affiliations:** ^1^Departamento de Zootecnia, Universidade Federal de Viçosa, Viçosa, Brazil; ^2^Department of Animal Sciences Dairy Cattle Nutrition, Washington State University, Pullman, WA, United States; ^3^Department of Animal Sciences, Colorado State University, Fort Collins, CO, United States

**Keywords:** livestock, poultry, milk production, minerals, vitamins

With the consolidation of production facilities, development and implementation of feed and growth technologies, and improvements in genetics, production efficiency of meat, milk, egg, and fiber has dramatically increased. Improved production efficiency has allowed for a more consistent and reliable supply of animal sourced food and fiber products at a low cost to the consumer. With improvements in animal genetics and use of growth technologies, the ability to supply the appropriate nutrients to animals to optimize growth efficiency is essential. Therefore, understanding the nutrient requirements for production animals is critical, not only to enhance growth efficiency, but to optimize the use of resources.

The following group of publications was assembled to discuss our current understanding of nutrient requirements for animals used for meat, milk, egg, and fiber production. It would be impossible to cover the most recent research for every nutrient required for all animals used for food and fiber production. Therefore, the following publications have been assembled to cover a variety of animal species (cattle, goats, pigs, chickens, and sheep) while focusing on a specific nutrient or modeling approach to define requirements.

Understanding factors that influence milk production in beef cows is critical for determining the nutrient requirements of cows. Researchers have attempted to use modeling techniques to estimate nutrient requirements for milk production in beef cattle. However, numerous factors can impact nutrient requirements of livestock. Therefore, a research group from the Universidade Federal de Viçosa in Brazil evaluated previously published non-linear models to predict the lactation curve of beef cows according to parity order under grazing conditions. Using 36 pregnant Nellore beef cows spanning three parity classifications (nulliparous, primiparous, and multiparous), the authors were able to determine that parity influences both milk yield and milk composition. Furthermore, the authors were able to determine which published milk yield prediction equations most accurately predicted milk yield for each parity classification of Nellore beef cows.

Milk production is an energy demanding process. Therefore, appropriate nutrient delivery to lactating livestock is critical to prevent a variety of metabolic disorders. Due to the increase in energy demand during lactation, diets containing elevated concentrations of grain are fed. In ruminants, feeding high-grain diets for an extended period of time can cause subacute ruminal acidosis and damage to the rumen epithelium, ultimately decreasing milk production. To address this problem, researchers from Nanjing Agricultural University in China examined the influence of adding a buffering agent to the diet of lactating goats fed a high grain-based diet. The researchers reported that adding a buffering agent to the diet increased milk protein content and amino acid flow to the mammary gland.

Minerals and vitamins are essential for proper growth, immune function, cellular homeostasis, reproduction, and carbohydrate, protein, and lipid metabolism in livestock and poultry. Therefore, supplementation of minerals and vitamins is essential when basal dietary ingredients do not meet the requirements of the animal or the basal diet contains elevated concentrations of a known antagonist. Research from Šperanda et al.'s laboratory indicates supplemental Se source and the addition of zeolite to the diet alters Se metabolism and immune and antioxidant status in pigs. Furthermore, using RNA-seq technology, Zhang et al. demonstrated that supplementing folic acid to broilers alters genes belonging to pathways related to hepatic lipogenesis which may ultimately reduce fat accumulation in the animal.

The final three publications focus on determining and/or re-evaluating the maintenance and growth requirements for sheep and goats. Mendes et al. fed Dorper × Santa Ines lambs for various rates of gain and conducted carcass dissections to determine NEm and NEg. Results from their experiment indicate the NEm for Dorper × Santa Ines lambs is similar to the current recommendations by the international committees; however, requirements for gain are lower. Finally, meta-analyses conducted by Santos et al. and Souza et al. were used to develop microbial crude protein synthesis prediction equations for both goats and sheep. They also determined that sex does not impact the protein requirements for maintenance and the efficiency of use of metabolizable protein.

The publications in this issue focus on better understanding the nutrient requirements for livestock and poultry. The results presented will help to adjust current prediction equations for estimating nutrient needs for livestock and poultry. Furthermore, these data help to provide a better understanding of the impact of specific nutrients on carbohydrate, protein and lipid metabolism.

## Author contributions

All authors listed have made a substantial, direct, and intellectual contribution to the work and approved it for publication.

